# Myocardial T2 mapping for improved detection of inflammatory myocardial involvement in acute and chronic myocarditis

**DOI:** 10.1186/1532-429X-16-S1-O63

**Published:** 2014-01-16

**Authors:** Rocio Hinojar, Lucy Foote, Eduardo Arroyo Ucar, Darius Dabir, Bernhard Schnackenburg, David M Higgins, Tobias Schaeffter, Eike Nagel, Valentina Puntmann

**Affiliations:** 1 Cardiovascular Imaging Department, King's College London, London, UK, London, UK; 2 Philips Healthcare, London, UK

## Background

Cardiac magnetic resonance (CMR) increasingly adds to clinical conformation of the diagnosis in patients with suspected myocarditis. The proposed Lake Louise Consensus Criteria ("any-two" approach) can separate from chronic forms of myocardial inflammation. However, both global enhancement ratio (GRE) and T2-weighted imaging are underutilized, due to poor reproducibility and high susceptibility to artefacts. T1 and T2 mapping by CMR projects tissue-dependent relaxation times. T2 mapping has been recently proposed as a robust and accurate technique to identify areas of focal myocardial oedema. Our aim was to investigate the value of quantitative T2 values in discrimination between health and disease, and separation between active/acute myocarditis and chronic convalescent stage of the disease.

## Methods

Twenty-four patients with acute presentation of viral myocarditis and twenty-three subjects in clinical convalescence were recruited. Thirty-three healthy subjects were served as controls. All subjects underwent CMR study for routine assessment of myocardial oedema, function and scar by at 3-Tesla scanner. T2 values were acquired in midventricular short-axis slice (mSAX) using GraSE sequence. We examined regional T2 values in patients and controls. T2 values are presented as an average of the six segments per mSAX. Secondly we investigated the differences between visually involved and remote myocardium (involved myocardium = areas by LGE, remote = areas with no LGE)

## Results

Patients with acute myocarditis and chronic myocarditis showed significantly raised T2 values [controls vs. acute vs. chronic myocarditis, T2 myocardium (msec): 48 ± 3 vs. 59 ± 9 vs. 53 ± 5, p < 0.0001). Compared to controls, T2 values in remote myocardium were significantly different for acute myocarditis only (T2, msec: 48 ± 3 vs. 51 ± 5 vs. 49 ± 2, p < 0.01), whereas T2 values in involved myocardium differed between all groups (T2, msec: 48 ± 3 vs. 69 ± 13 vs. 57 ± 8, p < 0.0001]. Using T2 values of complete mSAX or involved were identified as the independent discriminators between active and chronic myocarditis. T2 values were concordant with T2 oedema ratio (T2 involved, r = 0.42, p < 0.0001) and with native T1 (r = 0.55, p < 0.001).

## Conclusions

We demonstrate that quantitative T2 values are increased in patients with myocarditis. We further demonstrate that average mSAX and involved T2 values can discriminate between acute and chronic stage of the disease.

## Funding

We would like to acknowledge Department of Health via the National Institute for Health Research (NIHR) comprehensive Biomedical Research Centre award to Guy's & St Thomas' NHS Foundation Trust in partnership with King's College London and King's College Hospital National Health Service Foundation Trust. Dr. Rocio Hinojar was supported by the Spanish Society of Cardiology.

